# *Mycobacterium tuberculosis* Zinc Metalloprotease-1 Assists Mycobacterial Dissemination in Zebrafish

**DOI:** 10.3389/fmicb.2016.01347

**Published:** 2016-08-29

**Authors:** Mani H. Vemula, Raghavender Medisetti, Rakesh Ganji, Kiran Jakkala, Swetha Sankati, Kiranam Chatti, Sharmistha Banerjee

**Affiliations:** ^1^Department of Biochemistry, School of Life Sciences, University of HyderabadHyderabad, India; ^2^Biology Department, Dr. Reddy’s Institute of Life SciencesHyderabad, India

**Keywords:** tuberculosis, *Mycobacterium tuberculosis*, Zinc metalloprotease-1, zebrafish, mycobacterial dissemination

## Abstract

Zinc metalloprotease-1 (Zmp1) from *Mycobacterium tuberculosis* (*M.tb*), the tuberculosis (TB) causing bacillus, is a virulence factor involved in inflammasome inactivation and phagosome maturation arrest. We earlier reported that Zmp1 was secreted under granuloma-like stress conditions, induced Th2 cytokine microenvironment and was highly immunogenic in TB patients as evident from high anti-Zmp1 antibody titers in their sera. In this study, we deciphered a new physiological role of Zmp1 in mycobacterial dissemination. Exogenous treatment of THP-1 cells with 500 nM and 1 μM of recombinant Zmp1 (rZmp1) resulted in necrotic cell death. Apart from inducing secretion of necrotic cytokines, TNFα, IL-6, and IL-1β, it also induced the release of chemotactic chemokines, MCP-1, MIP-1β, and IL-8, suggesting its likely function in cell migration and mycobacterial dissemination. This was confirmed by Gap closure and Boyden chamber assays, where Zmp1 treated CHO or THP-1 cells showed ∼2 fold increased cell migration compared to the untreated cells. Additionally, Zebrafish-*M. marinum* based host–pathogen model was used to study mycobacterial dissemination *in vivo*. Td-Tomato labeled *M. marinum* (TdM. marinum) when injected with rZmp1 showed increased dissemination to tail region from the site of injection as compared to the untreated control fish in a dose-dependent manner. Summing up these observations along with the earlier reports, we propose that Zmp1, a multi-faceted protein, when released by mycobacteria in granuloma, may lead to necrotic cell damage and release of chemotactic chemokines by surrounding infected macrophages, attracting new immune cells, which in turn may lead to fresh cellular infections, thus assisting mycobacterial dissemination.

## Introduction

Tuberculosis (TB) is a highly contagious, chronic, airborne infection caused by bacilli belonging to genus mycobacteria, primarily, *Mycobacterium tuberculosis* (*M.tb*) (O’Garra et al., 2013). TB continues to pose a major health challenge worldwide, especially as a co-epidemic with HIV (Global Tuberculosis Report, 2015). India is one of the worst hit countries accounting for 23% of total global TB cases resulting in 2.2 lakhs mortality annually as estimated by World Health Organization (WHO) (Global Tuberculosis Report, 2015). The TB-causing bacilli, *M.tb* enter the host through aerosol route and are engulfed by alveolar macrophages, polymorphonuclear neutrophils and type 2 pneumocytes by phagocytosis (Smith, 2003). In most of the cases, the infection does not result in disease as the bacillus has evolved evasion strategies to live in balance with the immune response, thus remaining latent for decades (Babalola, 2015). A hallmark of immune reaction to TB bacilli is the formation of granuloma, by which host attempts to contain the infection (Guirado and Schlesinger, 2013). The granuloma is an aggregate of various immune cells, such as, macrophages, dendritic cells, and lymphocytes whose function depends on the cytokine environment generated due to TB infection (Ordway et al., 2006). Some of the infected cells undergo necrosis and create an acellular central zone where TB bacilli persist within granuloma. This necrotic zone eventually disintegrates in certain immunocompromised hosts, triggered by a mechanism still unknown, causing reactivation (Silva Miranda et al., 2012; Guirado and Schlesinger, 2013).

A significant aspect of the pathogenesis of virulent mycobacteria, like *M.tb*, is the ability to modulate cell death pathways where apoptotic cell death is considered bactericidal and necrotic cell death possibly assists bacterial dissemination and transmission (Chen et al., 2008; Gan et al., 2008; Divangahi et al., 2009; Behar et al., 2010). In this regard, several secretory proteins of *M.tb* have been implicated in either initial establishment of lung infections or extrapulmonary dissemination. To exemplify, ESAT-6 limits macrophage responses by inhibiting signaling from Toll-like receptor-2 (TLR-2) and causes phagosomal membrane lysis, thus helping establishment of infection, while HbhA, a glycoprotein, found on *M.tb* surface and also in culture filtrates, is not required for initial infection, but has possible role in dissemination to extrapulmonary regions (Pethe et al., 2001; de Jonge et al., 2007; Pathak et al., 2007). Yet other proteins, unique to mycobacteria genus, like PE25/PPE41 protein complex, has been shown to induce necrosis in macrophages and speculated to have a role in dissemination and disease reactivation (Tundup et al., 2014).

An interesting group of secreted proteins is extracellular Zinc-metalloproteases. These have been documented to contribute to the virulence of pathogenic bacteria by a variety of mechanisms. Several of these are established exotoxins and virulence factors, such as metalloprotease from *V. cholerae* O1 serotype (Finkelstein and Hanne, 1982; Hase and Finkelstein, 1993) or enterotoxin from *Bacteroides fragilis* (Obiso et al., 1995). In local bacterial infections, such as by *Staphylococcus* or *Pseudomonas* (keratitis, dermatitis) or by *Streptococcus* (pneumonia), the secreted metalloproteases cause necrotic or hemorrhagic tissue damage through digestion of structural components of the ground substance, enhancing vascular permeability permitting bacterial dissemination (Miyoshi and Shinoda, 2000). Similarly, clostridial neurotoxins are Zinc-metalloproteases that act by specifically cleaving a synaptic vesicle membrane or the presynaptic plasma membrane protein (Hayashi et al., 1994).

So far, there are evidences of three Zinc-metalloproteases from *M.tb* in the culture filtrate, namely, Rv2869 (Rip), Rv2467 (pepN) and Rv0198c (Zmp1). Rv2869 (Rip), a secretory metalloprotease has been shown to regulate intramembrane proteolysis and proteolytic degradation of anti-sigma substrates like RsdA controlling the SigD mediated transcriptional regulation in mycobacteria during stationary phase and hypoxia (Raman et al., 2004; Calamita et al., 2005) and thereby playing a role in mycobacterial virulence (Makinoshima and Glickman, 2005; Sklar et al., 2010). Rv0198c (Zmp1), supported by deletion mutant studies, was implicated in suppression of inflammasome activation by inhibiting caspase-1 activity and phagosome maturation, leading to decreased pathogen clearance suggesting a key role of Zmp1 during *M.tb* pathogenicity (Master et al., 2008; Johansen et al., 2011). In a recent study from our lab, we identified and characterized purified Zmp1 as a mycobacterial antigen that is secreted during granuloma-like stress conditions and generated Th2 cytokine microenvironment upon exogenous treatment of PBMCs, which was supported by recording specific and robust humoral response in a large cohort of TB patients (Vemula et al., 2016). Interestingly, the purified Zmp1 protein was earlier shown to cleave synthetically generated neuropeptides (Petrera et al., 2012). With *M.tb* Zmp1 reported as a virulence factor holding the properties of immunomodulation, high immunogenicity and proteolysis of synthetic neuropeptides, we further extended the study on the other possible roles of Zmp1 in the pathogenesis of *M.tb*.

In this study, we observed that exogenous treatment by high concentration of purified recombinant Zmp1 possibly induced necrotic cell death of THP1 cells, while sub-toxic levels promoted cell migration and release of chemotactic chemokines. In agreement with the reports that necrotic cell death during TB infection is an imperative precondition for bacterial dissemination, we further established, using zebrafish infected with fluorescently labeled *Mycobacterium marinum* that exogenous (secretory) Zmp1 helped in dissemination of mycobacteria. With this study, along with the earlier observations from our laboratory and others, *M.tb* Zmp1 has emerged as a multi-faceted protein which can be further explored as either a vaccine candidate, biomarker or anti-mycobacterial target.

## Materials and Methods

The cell lines used were Chinese Hamster Ovary (CHO) cell line and THP-1 Monocyte leukemia cell line (Cat#TIB-202, ATCC). Bacterial strains used were *M.tb* H37Rv and *Mycobacterium marinum*. Cell culture media and fetal bovine serum were purchased from Himedia, India. Casein was obtained from Sigma-Aldrich, USA. BD OptEIA^TM^ capture ELISA sets and eBioscience Human cytokine ELISA Ready-Set-Go kits were used. MTT, DMSO, and other chemicals were purchased from HiMedia, India. All plasticware and glassware were obtained from Tarsons, India; Eppendorf, Germany, and Corning, USA. All procedures for zebrafish experimentation were as per guidelines published by the National Institutes of Health USA for care and use of zebrafish (Wilson et al., 2009). Zebrafish husbandry and all experimental procedures were part of a protocol approved by an Institutional Biosafety Committee (IBSC Approval No. DRILS/IBSC/2013/06). Indigenous wildtype male and female adult zebrafish were used for this study (obtained from Vikrant Aquaculture, Mumbai, India and further generations bred in our laboratory). Fish were maintained in a custom-built recirculation system with polysulphone housing tanks containing purified water (Millipore ELIX system grade) with 200 mg/L sea salt at 28°C under a 14:10 h light and dark cycle (Westerfield, 2000). Fish were fed three times daily with live hatched brine shrimp and dry food. Zebrafish were spawned in a Mini Mass Embryo Production System (MEPS^®^ – Aquatic Habitats). Fertilized eggs were collected and maintained in either tank water or in embryo medium. Larval stages were raised in static tanks and fed with paramecia or spirulina from 5 dpf (day post-fertilization) to 15 dpf and then weaned onto Artemia as food.

### Maintenance of Cell Lines

The THP-1 cell lines were maintained in RPMI 1640 media supplemented with 10% FBS (South American origin, Gibco) and incubated at 37°C with 5% CO_2_. The media was changed when the confluency of the cells reached 90%. The CHO cell lines were maintained in RPMI media supplemented with 10% FBS and incubated at 37°C with 5% CO_2_. The media was changed when the cells reached 90% confluency, the cells were trypsinized with the Trypsin-EDTA (Sigma) and washed with phosphate buffered saline pH 7.4 (PBS) (Ganji et al., 2016b).

### Growth and Maintenance of Mycobacterial Culture

*Mycobacterium marinum* transformed with pTEC27 harboring Td-Tomato (TdM. marinum) was plated on 7H10 agar media (Hi-media, India) supplemented with 10% Oleic acid, Albumin, Dextrose and Catalase (OADC, Hi-Media, India) in presence of Hygromycin (50 μg/mL). The plate was incubated at 30°C until colonies appeared. The colonies were picked into the 7H9 broth supplemented with 10% OADC, Hygromycin (50 μg/mL) and the broth culture was incubated at 30°C at 180 rpm until the OD_600__nm_ reached 0.8–1. The culture was checked for any contamination and single suspension of bacteria was obtained as described earlier (Ganji et al., 2016a).

### Growth and Maintenance of Bacterial Cultures

pET28a (+) plasmid carrying *zmp1* (*Rv0198c*) (Vemula et al., 2016) or *Rv0047c* genes were transformed into *Escherichia coli* BL21-DE3 cells. A single bacterial colony carrying plasmid were inoculated into LB medium containing kanamycin (34 μg/mL) and were incubated overnight at 37°C with vigorous shaking (180 rpm). The next day, secondary cultures were set up in LB media with 2% inoculum of overnight grown primary cultures and were incubated at 37°C with vigorous shaking (180 rpm). When OD at 600 nm reached to 0.6, the cultures were induced with 0.5 mM β-D-Isopropyl thiogalactoside (IPTG) kept for 4 h at 37°C. The bacterial pellets were harvested and rZmp1 and rRv0047c proteins were purified using similar methodology as described previously (Vemula et al., 2016). The proteins were made endotoxin-free using polymyxin B agarose beads (Sigma Aldrich). The residual endotoxin levels were quantified using Pierce^TM^ LAL Chromogenic Endotoxin Quantitation Kit. The residual endotoxin (LPS) levels in the purified rZmp1 and rRv0047c were observed to be 0.046 ± 0.001 EU/mL and 0.043 ± 0.001 EU/mL, respectively. The proteolytic activity of rZmp1 using casein as substrate was performed as described earlier (Vemula et al., 2016). Heat-denaturation of rZmp1 or rRv0047c was performed by heating the purified proteins at 100°C for 15 min followed by snap-freezing on ice. Native and heat denatured rZmp1 were later checked for caseinase activity (**Supplementary Figure S1A**).

### MTT (3-(4, 5-Dimethylthiazol-2-yl)-2, 5-Diphenyltetrazolium Bromide) Assay

0.2 × 10^5^ cells (THP-1 and CHO cell lines) per well were seeded in 200 μL media into a 96 well plate and incubated overnight at 37°C and 5% CO_2_. The cells were incubated exogenously with varying concentrations of purified rZmp1 protein ranging from 5 nM to 2 μM for 24 h. Similarly, THP-1 cells were exogenously treated with rRv0047c at concentrations 50 and 100 nM. The cell viability was assessed using MTT assay (**Supplementary Figures S1B** and **S2**). Twenty microliter of 5 mg/mL MTT solution was added to each well and incubated the plate at 37°C, 5% CO_2_ for 4 h. The plate was centrifuged at 3000 rpm for 5 min and excess media (150 μL) was removed. The formazan crystals were dissolved in 150 μL of DMSO. Absorbance was measured at 540 nm using multi-well plate reader.

### Gap Closure Assay

Chinese Hamster Ovary cells were allowed to reach >90% confluence in 35 mm cell culture dish. For the assay, prepared 10 ml of base media with 50 nM of rZmp1 protein and filter sterilized. Meanwhile, a line was drawn with a marker on the bottom of the dish. Using a sterile tip, three separate wounds were scratched through the cells moving perpendicular to the line drawn before. Cells were rinsed gently with PBS and replaced with 1.5 mL of media either containing rZmp1 protein or only buffer (control set). Pictures were taken using phase contrast at 10× magnification at 0, 16, 24, and 48 h. The same was repeated with the control set without rZmp1. The gap closure was measured in both control set and rZmp1 containing set using ImageJ software (Liang et al., 2007).

### Boyden Chamber Assay

24-well tissue culture plate containing cell culture inserts with polycarbonate membrane of 8 μm pore size were taken. 200 μL of media with 0.4 × 10^5^ cells without rZmp1 was added to upper chamber of cell insert and 750 μL of media without cells containing only 50 nM of endotoxin-free rZmp1 protein or rRv0047c protein was added to lower chamber. Corresponding heat-denatured protein controls were also used. The plate was then placed at 37°C with 5% CO_2_ for 24 h. The cells in the upper and lower chamber were collected separately by centrifuging at 200–300 *g* for 5 min. The cell numbers were then determined by Trypan blue exclusion assay using hemocytometer.

### Capture ELISA Protocol

Capture ELISA for cytokine measurements was performed as per the manufacturer’s instructions. Briefly, 50 μL of diluted capture antibody was added to the wells of ELISA/RIA compatible 96-well plate and kept overnight at 4°C. Followed by three PBS-T (PBS containing 0.05% Tween-20) and one PBS washes. The coated plate was blocked using 3% bovine serum albumin in PBS at 37°C for 1 h. After blocking, washes were performed with PBS-T and PBS as mentioned before. Fifty microliter sample was added and incubated at 37°C for 2 h which was followed by five PBS-T and one PBS washes. Then 50 μL of diluted detection antibody along with streptavidin-HRP conjugate was added and incubated at 37°C for 1 h, followed by 7 PBS-T and one PBS washes. 3,3′,5,5′-Tetramethylbenzidine (TMB) was used as substrate according to manufacturer’s directions. Dispensed 100 μl into each well in dark and incubated at RT (5–30 min) for color development. The reaction was stopped using 100 μl of 2N H_2_SO_4._ Read the optical density (OD) for each well with a microplate reader set to 450 nm.

### Lactate Dehydrogenase (LDH) Assay

The lactate dehydrogenase activity was determined by measuring the decrease in the absorbance at 340 nm resulting from the oxidation of NADH. The reaction mix consisted of 0.66 mM NADH and 3 mM Sodium pyruvate in 0.2M Tris-HCl, pH 7.3. To 100 μL of reaction mix 20 μL of culture supernatant of THP-1 cells treated with or without rZmp1 was added and monitored the change in NADH levels at 340 nm.

### Propidium Iodide Staining for Flow Cytometry

THP-1 cells were treated with or without rZmp1 (500 nM and 1 μM) for 24 h. After incubation, the media was removed, added prewarmed RPMI complete media containing 40 μg/mL of propidium iodide (PI) for 15 min at room temperature (RT) in dark. After staining, the cells were washed twice in PBS and then fixed with 3% paraformaldehyde at RT for 20 min. Then the cells were washed twice with PBS followed by RNase (50 μg/mL) treatment at 37°C for 15 min. The cells were then washed with PBS and resuspended in PBS for flow cytometry analyses.

### TdM. Marinum Dissemination Assay in Zebrafish

Fluorescently labeled TdM. marinum was injected at 200 CFU (colony forming unit) with or without rZmp1 [25 to 100 nM native or 100 nM heat-denatured protein (HD-rZmp1)] or non-specific, recombinant mycobacterial protein, rRv0047c (100 nM native or 100 nM heat-denatured protein (HD-rRv0047c)) into yolk sac of 1 dpf (day post fertilization) Zebrafish. Images were taken at the 0 day of injection and on the 5th day after injection of TdM. marinum using fluorescent microscopy. Fishes were scanned for the presence of TdM. marinum in the whole organism and images were taken accordingly. Fluorescence intensities of TdM. marinum in the fish were quantified using ImageJ software. Total fluorescence was calculated by taking count of all the regions in a whole fish (Benard et al., 2012). Dissemination of TdM. marinum toward tail region was calculated by two parameters and plotted: (1) Relative percentage of fish with TdM. marinum disseminated to tail region = (Number of fish with TdM. marinum disseminated to tail region / total number of fish injected with TdM. marinum in the category) × 100; (2) Dissemination to tail region = (Fluorescence intensity in tail region/total fluorescence intensity in whole fish) × 100. Survival of Zebrafish for the rZmp1was evaluated by injecting the fish with rZmp1 (ranging from 25 to 100 nM or 100 nM heat-denatured rZmp1) alone without any TdM. marinum and monitored the fish over the course of 5 days.

### Graphs and Statistical Analyses

SigmaPlot software version 11.0.0.77 (Systat Software, Inc., USA) was used for different statistical analyses. For cytokine data and cell migration data, One-way ANOVA was performed with Holm–Sidak multiple pair-wise comparison method and the threshold for significance was set at *p* < 0.05. The error bars represent the ± standard deviation (SD) from the mean of at least three independent experiments. For statistical analyses of mycobacterial dissemination data, One-way ANOVA on ranks was performed with Dunn’s method for pair-wise comparison method for comparing more than two groups or Students *t*-test with Mann–Whitney Rank sum test was performed for comparing two groups. The threshold for significance was set at *p* < 0.05. They were represented as box plots using SigmaPlot. Within the plots, the upper quartile of the box represents the 75th percentile and the lower quartile for the 25th percentile. The line inside the box represents the median. The whiskers arising from either side of the upper half and the lower half of the box correspond to 1.5 times the interquartile range (IQR) (Benjamin et al., 2013; Vemula et al., 2016). Any datum to the further extreme of the whiskers is termed as outlier.

## Results

### Higher Concentrations of Exogenous rZmp1 Treatment Caused Necrotic Cell Death

**FIGURE 1:**
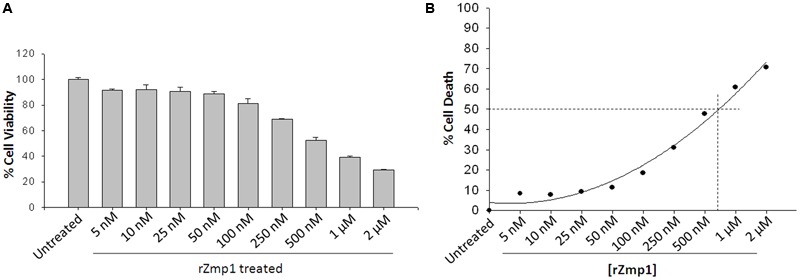
**Exogenous treatment with rZmp1 at higher concentrations caused cell death with IC_50_ of ∼550 nM in the conditions studied. (A)** Bar graph represents the percentage THP-1 cell viability upon exogenous treatment with varying concentrations of rZmp1. **(B)** Scatter plot represents the percentage THP-1 cell death upon exogenous treatment with varying concentrations of rZmp1. The dotted lines depict the concentration of rZmp1 (∼550 nM) at which there was 50% cell death (IC_50_). All the experiments were performed more than three times. The error bar represents standard deviation from mean.

**FIGURE 2:**
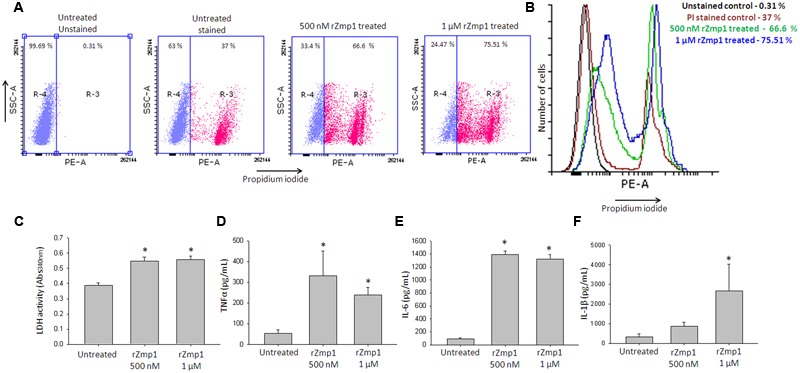
**Exogenous treatment of THP-1 with rZmp1 induced necrotic cell death. (A)** The dot plots show the propidium iodide staining for the conditions, Untreated unstained, Untreated stained, 500 nM rZmp1 and 1 μM rZmp1 treated cells. The THP-1 cells were either only buffer treated or rZmp1 treated (500 nM and 1 μM) for 24 h. Propidium iodide staining followed by flow cytometry were used to evaluate necrosis of THP-1 cells. **(B)** The histogram plot depicts the extent of PI staining upon rZmp1 treatment. **(C)** Bar graphs represent lactate dehydrogenase activity of the culture supernatants of THP-1 cells treated either with buffer or rZmp1. **(D–F)** Bar graph represents the levels of cytokines, **(D)** TNFα, **(E)** IL-6 and **(F)** IL-1β determined in the culture supernatants of rZmp1 treated cells, using capture ELISA method. All the experiments were performed at the least three times. The error bars represent standard deviation from mean. ^∗^represents statistical significance with *p* < 0.05.

Secreted Zinc metalloproteases across pathogenic bacteria are known to act as toxins causing a range of pathological effects by virtue of their proteolytic activities, quite often causing necrotic tissue damage (Hase and Finkelstein, 1993). To begin with, we checked for the toxicity of the recombinant Zmp1 (rZmp1) on human monocyte leukemia cell line THP-1 and Chinese Hamster Ovary (CHO) cell lines (**Figure 1**; **Supplementary Figure S1B**). THP-1 monocytes or CHO cells were treated with varying concentrations of endotoxin-free rZmp1 and assayed for cell death using MTT assay. It was observed that though rZmp1 protein was not toxic at lower concentrations of 50 nM but was toxic at high concentrations at 24 h (**Figure 1A**). The IC_50_ calculated for Zmp1 cytotoxicity on THP-1cells was ∼550 nM (**Figure 1B**). We further observed that the toxicity at higher concentrations was due to necrotic mode of cell death as determined by flow cytometry using Propidium Iodide (PI) staining of the rZmp1 treated cells and Lactate Dehydrogenase (LDH) assay of the supernatants of the rZmp1 treated cells (**Figures 2A–C**). It was observed that rZmp1 treated cells at both 500 nM and 1 μM concentrations showed increased PI stained population suggesting cell death due to necrotic damage (**Figures 2A,B**). The 500 nM and 1 μM rZmp1 treated cells showed 66.6 and 75.51% PI positive population, respectively (**Figures 2A,B**). Upon LDH assay of the culture supernatants of the rZmp1 treated and untreated THP-1 cells, it was observed that culture supernatants of rZmp1 (both 500 nM and 1 μM) treated cells showed increased LDH activity (500 nM rZmp1: 0.548 ± 0.03 Abs_340_; 1 μM rZmp1: 0.558 ± 0.02 Abs_340_) as compared to untreated controls (0.388 ± 0.02 Abs_340_) suggesting the mode of cell death to be necrotic in nature (**Figure 2C**). We further checked the levels of cytokines, TNFα, IL-6, and IL-1β, which are implicated in necrosis (Kaczmarek et al., 2013; Moriwaki et al., 2015), in the culture supernatants of the THP-1 cells treated with rZmp1 (500 nM and 1 μM) (**Figures 2D–F**). The levels of cytokines as measured by ELISA were TNFα (Untreated: 54.82 ± 14.75 pg/mL; 1 μM rZmp1 treated: 239.46 ± 35.31 pg/mL; *p* < 0.05) (**Figure 2D**), IL-6 (Untreated: 91.93 ± 15.92 pg/mL; 1 μM rZmp1 treated: 1318.47 ± 74.23 pg/mL; *p* < 0.05) (**Figure 2E**) and IL-1β (Untreated: 328.19 ± 168.89 pg/mL; 1 μM rZmp1 treated: 2665.31 ± 1366.98 pg/mL; *p* < 0.05) (**Figure 2F**), respectively. As anticipated there was significant increase in the secretions of these cytokines by THP-1 into the culture supernatants upon rZmp1 exposure as compared to untreated cells further pointing that exogenous treatment of rZmp1 at higher concentrations, possibly, induced necrosis.

### rZmp1 Promoted Cell Migration in THP-1 Cells and Induced Secretion of Chemotactic Chemokines

**FIGURE 3:**
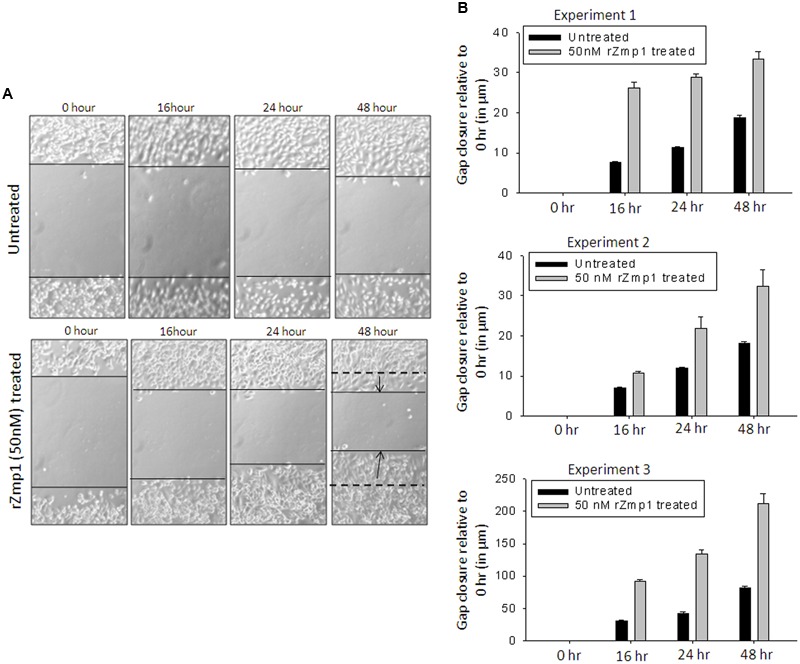
**rZmp1 treatment helped CHO cell migration as observed by Gap closure assay. (A)** Representative microscopic image from experiment 1 showing CHO cell migration as function of time upon 50 nM rZmp1 exogenous treatment. Monolayer of CHO cells were grown in 35 mm cell culture dish. A gap was introduced using a sharp tip and the cells were either only buffer treated (Upper panel) or 50 nM rZmp1 treated (lower panel). The extent of gap closure was monitored microscopically at regular intervals (0, 16, 24, and 48 h). **(B)** Bar graphs represents the distance of gap closure in μm (micrometers) relative to 0 h calculated using ImageJ software at regular intervals from three independent experiments. The error bars represent the standard deviation from mean.

**FIGURE 4:**
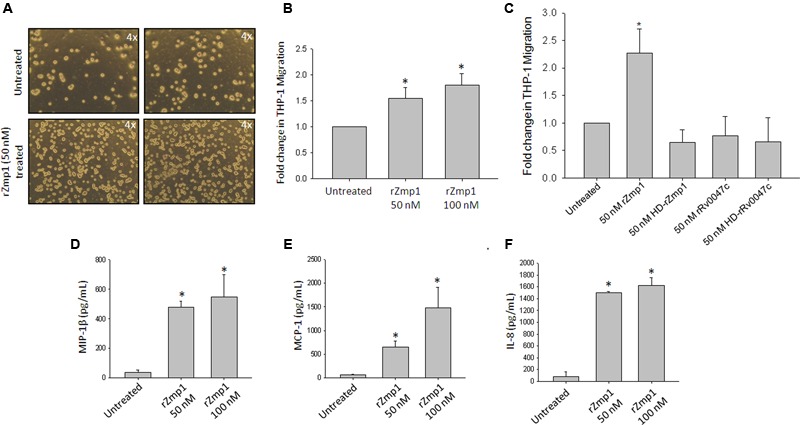
**Boyden chamber migration assays with rZmp1 treated and untreated THP-1 cells. (A)** Representative microscopic image showing THP-1 cells migrated to the lower chamber upon 50 nM rZmp1 treatment. THP-1 cells were seeded into the upper chamber of the Boyden chamber and the lower chamber contains media along with buffer control or 50 nM rZmp1 and incubated for 24 hr. 50 nM rZmp1 containing lower chamber showed more THP-1 cells migrated from upper chamber. **(B,C)** Bar graph represents the fold change in THP-1 migration in untreated, rZmp1 (50, 100 nM native or 50 nM HD-rZmp1) or rRv0047c (50 nM native or 50 nM HD-rRv0047c) treated cells. The number of cells migrated to the lower chamber were counted using Trypan Blue live cell staining assay and plotted the increase in fold change in THP-1 cells migrated from upper chamber to lower chamber. (D-F) The culture supernatants of rZmp1 (50 and 100 nM) treated cells were assayed for chemokines implicated in cell migration or chemotaxis, **(D)** MIP-1β, **(E)** MCP-1 and **(F)** IL-8 using capture ELISA method. All the experiments were performed at the least three times. The error bars represent standard deviation from mean. ^∗^represents statistical significance with *p* < 0.05.

Necrotic cell death of the *M.tb* infected cells has been linked to mycobacterial dissemination (Molloy et al., 1994; Lee et al., 2011; Butler et al., 2012; Roca and Ramakrishnan, 2013). With high concentration of rZmp1 causing necrotic cell death, we proceeded to assess for the ability of rZmp1 to promote cell migration. For these assays, a sub-toxic level of rZmp1 concentration was used, so that excessive cell deaths can be avoided. The preliminary experiments were performed on CHO cells using the gap closure assays, which were further supported by Boyden chamber assays using THP-1 cells (**Figures 3** and **4**). Gap closure assays were performed using untreated or CHO cells treated with 50 nM rZmp1 (**Figure 3A**). Images were taken at different time points (0, 16, 24, and 48 h) and gap width was calculated and plotted (**Figure 3B**). Exogenous treatment of rZmp1 resulted in increased gap closure as compared to the untreated cells by 2.05 ± 0.47 folds at 48 hr (**Figure 3B**).

The ability of rZmp1 to promote cell migration was further confirmed by Boyden chamber assays using THP-1 monocyte cell line. In this assay, we preloaded the cells in the upper chamber with complete media and the lower chamber with media containing 50 and 100 nM endotoxin-free rZmp1, expecting that if rZmp1 indeed influenced migration of cells, the cells would move from the upper chamber to the lower chamber. It was observed that there was increased migration of THP-1 cells from the upper chamber to the lower chamber containing 50 and 100 nM rZmp1 as compared to control without rZmp1 (**Figures 4A,B**). The fold changes in the migration of THP-1 cell line in the presence of 50 and 100 nM rZmp1 were 1.55 ± 0.20 and 1.81 ± 0.21 fold, respectively as compared to untreated, exhibiting a dose dependent increase in the cell migration (**Figure 4B**). To further confirm that this property is specific to rZmp1 and not due to the residual endotoxin present in the recombinant protein preparations, we performed the same experiment with 50 nM heat-denatured rZmp1 (HD-rZmp1) and a randomly selected non-specific recombinant mycobacterial protein, rRv0047c [50 nM native or 50 nM heat-denatured protein (HD-rRv0047c)], which was purified and made endotoxin-free under conditions similar to that of rZmp1 purifications from recombinant protein expressing *E. coli*. It was observed that the migration of THP-1 cell line was specific to rZmp1 treatments and is not induced by rRv0047c or heat-denatured rZmp1 (**Figure 4C**). Taken together the gap closure assays and Boyden chamber assays confirmed involvement of rZmp1 in migration of cells.

We further checked if rZmp1 treatment of THP-1 cells resulted in release of chemotactic chemokines, such as MCP-1, MIP-1β and IL-8 that are known to recruit monocytes, neutrophils, memory T cells, and dendritic cells to the sites of infection (Taub et al., 1995; Gerszten et al., 1999; Bystry et al., 2001; Deshmane et al., 2009). THP-1 cells upon incubation with rZmp1 at 50 and 100 nM showed increased levels of all the three cytokines compared to the untreated cells (**Figures 4D–F**). The cytokine levels in the culture supernatants of THP-1 cells untreated or rZmp1 treated were, MCP-1 (Untreated: 57.97 ± 11.2 pg/mL; 50 nM rZmp1: 654.82 ± 118.97 pg/mL; 100 nM rZmp1: 1473.54 ± 443.12 pg/mL; *p* < 0.05) (**Figure 4D**), MIP-1β (Untreated: 38.51 ± 15.71 pg/ml; 50 nM rZmp1: 478.28 ± 42.06 pg/mL; 100 nM rZmp1: 548.58 ± 149.59 pg/mL; *p* < 0.05) (**Figure 4E**) and IL-8 (Untreated: 78.42 ± 79.08 pg/mL; 50 nM rZmp1: 1495.44 ± 25.88 pg/mL; 100 nM rZmp1: 1626.99 ± 130.99 pg/mL; *p* < 0.05) (**Figure 4F**).

Summing up the results so far, rZmp1 that induced necrotic cell death at higher concentrations, promoted cell migration and secretion of chemotactic chemokines by THP1 cells upon exogenous treatments at sub-toxic levels. One can thus speculate that upon causing necrosis, *M.tb* Zmp1 induces secretion of chemotactic chemokines that would assist migration of monocytes and dendritic cells to the necrotic site, causing fresh rounds of infections of these cells and thereby help dissemination of mycobacteria.

### rZmp1 Helped in Dissemination of Mycobacteria

**FIGURE 5:**
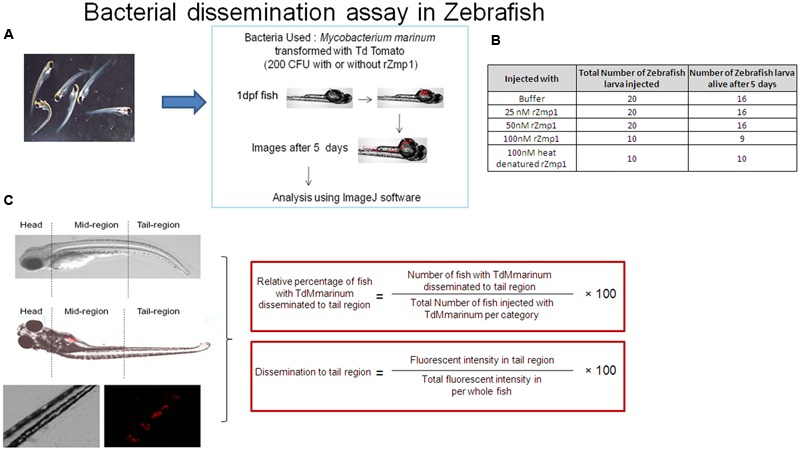
**Schematic representation of *M. marinum* dissemination assay in Zebrafish. (A)** 1 day post fertilization fish were taken for the experiments and injected with 200 CFU of Td-Tomato labeled *M. marinum* (TdM. marinum) along with either only buffer control or rZmp1 (25, 50, and 100 nM) into the yolk sac. The fish were then incubated in E3 medium for 5 days, followed by visualization of TdM. marinum under fluorescent microscope on 5th day to evaluate for bacterial dissemination to tail region. **(B)** Table represents the survival of Zebrafish for rZmp1 which was evaluated by injecting the fish with rZmp1 (ranging from 25 to 100 nM) alone without any TdM. marinum and monitored the fish over the course of 5 days. **(C)** To determine the bacterial dissemination to tail region, firstly each fish was divided into three regions, head, mid-region and tail-region as depicted in the figure. Fish were then scanned under fluorescence microscopy for the presence of TdM. marinum in the whole organism and images were taken accordingly. Fluorescence intensity of TdM. marinum present in the fish was quantified using ImageJ software. Total fluorescence was calculated by taking count of all the regions in a whole fish. Dissemination of TdM. marinum toward tail region was calculated by two parameters and plotted: (1) Relative percentage of fish with TdM. marinum disseminated to tail region and (2) Dissemination to tail region.

**FIGURE 6:**
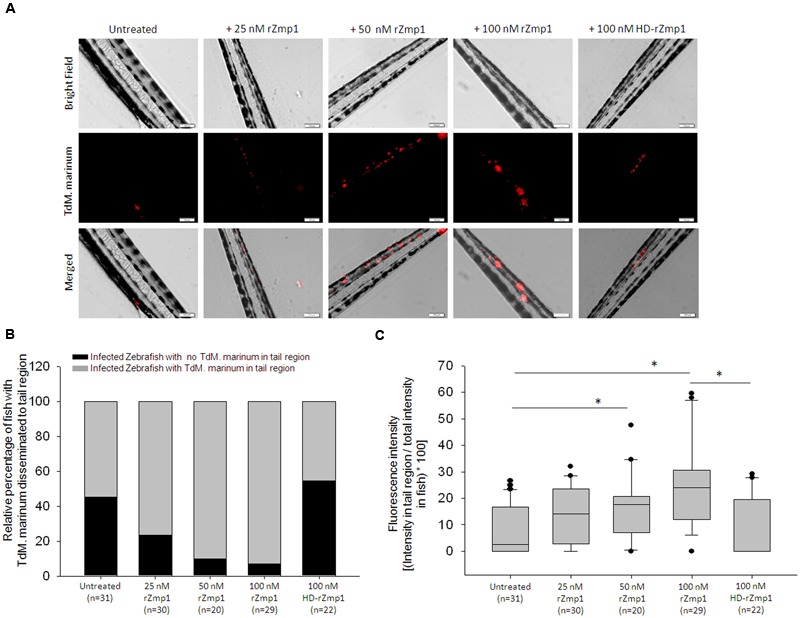
**Zmp1 helped in the dissemination of *M. marinum* in Zebrafish model. (A)** Representative images showing the presence of fluorescently labeled *M. marinum* (TdM. marinum; Red) in the tail region of the Zebrafish. One day post fertilization Zebrafish were injected with TdM. marinum either with buffer control (Untreated) or rZmp1 (25–100 nM) or heat-denatured rZmp1 (100 nM). The dissemination of TdM. marinum to the tail region was then observed under fluorescence microscope on 5th day post infection (refer materials and methods). **(B)** Bar graph representing the relative percentage of fish with TdM. marinum disseminated to tail region. **(C)** The box plot represents the percentage of dissemination of TdM. marinum to the tail region. All the experiments were performed more than three times. n represents the number of fish used per category. ^∗^represent statistical significance with *p* < 0.05.

To examine our hypothesis that Zmp1 indeed helped in mycobacterial dissemination, we used Zebrafish as a model organism which was infected with fluorescently labeled *M. marinum* (TdM. marinum) which harbored pTEC27 plasmid having Td-Tomato gene. Although Zmp1 of *M. marinum* is 84% similar to *M.tb* Zmp1, unlike *M.tb* Zmp1, *M. marinum* Zmp1 is predicted to be a cytosolic protein (Kapopoulou et al., 2011). Therefore, to simulate the presence of secreted Zmp1 in the vicinity of mycobacteria, *M. marinum* was either mixed with rZmp1or with buffer alone before injections into Zebrafish. We injected 200 CFU of TdM. marinum either mixed with buffer or with various concentrations of endotoxin-free rZmp1 (25, 50, 100, and 100 nM of heat-denatured rZmp1) into the yolk sac of 1 dpf (days post fertilization) Zebrafish larva (refer materials and methods) (**Figure 5A**). Initially toxicity of injected rZmp1 protein on Zebrafish was checked and found that rZmp1 was not toxic at any of the concentrations of 25, 50, and 100 nM used (**Figure 5B**). After injections with TdM. marinum with or without rZmp1, the fishes were monitored for 5 days and images were taken using fluorescence microscope on 0 day and fifth day (**Figure 5A**). Then we determined the spread of TdM. marinum from the site of injection to the tail region using two parameters (**Figure 5C**). Firstly, we calculated the percentage of fish in a category with fluorescence in the tail region and secondly, we measured the percentage of fluorescence intensity in tail region with respect to whole fish fluorescence intensity for each fish (**Figure 5C**). Using these parameters, we observed that there was an increase in dissemination of fluorescently labeled *M. marinum* to the tail regions from the site of injection in the infected Zebrafish with rZmp1 than in infected Zebrafish without rZmp1 in a dose dependent manner ranging from 25 to 100 nM (**Figure 6A**). It was observed that 54.84% of control fish showed fluorescent TdM. marinum in tail region (*n* = 31), while 76.67% of fishes injected with TdM. Marinum + 25 nM rZmp1 (*n* = 30), 90% of fishes injected with TdM. Marinum + 50 nM rZmp1 (*n* = 20) and 93.1% of fishes injected with TdM. Marinum + 100 nM rZmp1 (*n* = 29) showed fluorescent TdM. marinum in the tail region (**Figure 6B**). This indicated that rZmp1 helped in the dissemination of TdM. marinum from the site of injection to the tail region in a dose-dependent manner (**Figure 6B**). In order to confirm that the impact of rZmp1 on mycobacterial dissemination is specific, we heat inactivated rZmp1 by denaturing the protein at 100°C for 15 min. The 100 nM of heat-denatured rZmp1 was mixed with TdM. marinum and injected into Zebrafishes similar to other groups. Only 45.45% of fishes (*n* = 22) showed fluorescent TdM. marinum in the tail region, which was similar to untreated control group (**Figure 6B**).

We then measured the fluorescence intensity of TdM. marinum per fish and calculated the ratio of fluorescence intensity in the tail region with respect to the whole fish intensities (**Figure 5C**). With this, we observed that the control group infected with TdM. marinum without rZmp1 (Median: 2.66%; IQR: 0-16.55) showed significantly lesser percentage of fluorescence intensity in tail region compared to Zebrafish category infected with TdM. marinum+100nM rZmp1 (Median: 23.91%; IQR: 12.1 – 30.59) further confirming that rZmp1 helped in the enhanced dissemination of TdM. marinum from the site of injection to the tail region (**Figure 6C**). Further, Zebrafish group injected with TdM. Marinum + 100 nM heat-denatured rZmp1 (Median: 0%, IQR: 0 – 19.31) showed dissemination similar to the rZmp1 untreated control group (Median: 2.66%; IQR: 0-16.55) (**Figure 6C**).

The assays were also performed using rRv0047c (100 nM native or 100 nM heat-denatured), which was also used in Boyden chamber assay as a negative control. Dissemination of TdM. marinum in rRv0047 (*n* = 21) injected zebrafish groups were similar to that of control group (*n* = 17) as assessed by calculating the ratio of fluorescence intensity in the tail region with respect to the whole fish intensities (**Supplementary Figure S3**). These analyses confirmed the role of Zmp1 in mycobacterial dissemination in Zebrafish model.

## Discussion

With this study we added a new physiological function to the multi-faceted *M.tb* Zmp1. We observed that, exogenous treatment of macrophages with high concentrations resulted in necrotic cell death, at the same time, sub-toxic levels stimulated cells to migrate and release chemotactic chemokines. Together, these properties perhaps helped rZmp1 facilitating dissemination of mycobacteria, as observed in zebrafish infected with fluorescence labeled *M. marinum*.

Previous reports involving Zmp1 deletion mutants of *M.tb* advocated that Zmp1 is implicated in suppression of inflammasome activation. In these studies it was observed that deletion of Zmp1 resulted in improved pro-inflammatory response and antigen presentation (Master et al., 2008). Complementing these inferences, we also observed that exogenous treatment of PBMCs with rZmp1 biased the immune response toward Th2 (Vemula et al., 2016). It is well established that when Th2 wing of immune response gets activated it tends to suppress the pro-inflammatory Th1 response.

In this study, we observed that rZmp1 treatment induced secretions of cytokines TNFα, IL-6, and IL-1β (**Figures 2D–F**) which are well-documented to be associated in inducing necrotic cell death (Vanden Berghe et al., 2006; Kaczmarek et al., 2013; Moriwaki et al., 2015). In our conditions, we observed that rZmp1 exogenous treatment of THP-1 cells, induced a pro-inflammatory response showing necrotic cell damage. A possible explanation may be that in our earlier experiments (Vemula et al., 2016), we had observed that rZmp1 treatment of PBMCs and THP-1 cells resulted in release of high titers of IL-4, IL-6, and IL-1β, along with high TNFα. Concurrent high levels of TNFα with IL-4 increases the toxic potentiality of TNFα, causing necrotic tissue damage. This may result in liquefaction of TB granuloma, hence dissemination and relapse (Fenhalls et al., 2000; Rook, 2007). Also, TNFα at higher levels, in an autocrine or paracrine mode induces necrosis through RIP1/RIP3 kinase pathway (Roca and Ramakrishnan, 2013). In general, necrosis of the *M.tb* infected cells has been associated with the dissemination of bacilli and further infection of fresh cells. Careful evaluations with complementary experiments are further required to understand the exact mechanism of necrotic death caused by rZmp1.

Zinc containing metalloproteases are widely distributed among prokaryotes and eukaryotes. They are known to play a critical role in bacterial pathogenesis and in various physiological processes such as mammalian cells adhesion, migration, extracellular matrix remodeling and cell–cell interactions (Kearns et al., 2002). For example, Zmp1 protein of *Clostridium difficile* has been shown to degrade the fibrin network of fibroblasts and thus help in bacterial infection and dissemination (Cafardi et al., 2013). Pap6 of *Vibrio harveyi* and Hemagglutinin/protease from *Vibrio cholerae* have been shown to digest various ECM components such as fibronectin, collagen and gelatin (Booth et al., 1983; Finkelstein et al., 1983; Teo et al., 2003). Digestion of fibronectin network by proteases results in fibroblasts losing adherence property and thus may lead to cell migration (Cafardi et al., 2013). StcE, a secreted protease of *E. coli*, expressed during infection, contributes to virulence by affecting crucial neutrophil recruitment, migration and also oxidative burst production (Szabady et al., 2009). SslE, another extracellular zinc metalloprotease of *E. coli* through its mucinase activity help bacterial translocation through mucin-matrix allowing its penetration through the mucosal surface and reach host epithelium to make a niche inside the host (Nesta et al., 2014; Valeri et al., 2015). Based on the evidences that Zinc metalloproteases help in cell adhesion and migration, we sought to check the effect of rZmp1 on the cell migration. We employed both Gap closure assays and Boyden chamber assays on CHO cells and THP-1 cells, respectively and observed that rZmp1 treatments showed improved cell migration compared to control treatments (**Figures 3** and **4**). Though Gap closure assays using CHO cells confirmed the effect of rZmp1 on migration, Boyden chamber assays using THP-1 cells, which creates a concentration gradient between upper and lower chamber, could be inferred in the context of either cell migration and/or chemotaxis (Chen, 2005; Liang et al., 2007). We also learnt that rZmp1 treatment induced secretion of chemotactic chemokines, MCP-1, MIP-1β and IL-8 in the extracellular milieu (**Figures 4D–F**).

Hypothesizing that together with induction of necrosis, release of chemotactic chemokines and induction of cell migration, Zmp1 may influence dissemination of mycobacteria from the site of infection, we used Zebrafish to confirm the same. Zebrafish-*M. marinum* as a host-pathogen model has been used extensively for understanding tuberculosis pathogenesis, primarily due to the ease of genetic tractability and the visible transparency of Zebrafish larva during the early developmental stages (Ramakrishnan, 2013). Zebrafish, as a model, has been, so far successfully used to study the dissemination of various pathogens like *Mycobacterium, Salmonella, Burkholderia, Staphylococcus, Shigella*, and *Candida* (Torraca et al., 2014). *M. marinum*, the close relative of *M.tb*, can infect Zebrafish and is known to cause formation of granuloma structures similar to *M.tb* granuloma in humans. It can also cause latent infections in adult Zebrafish that can be reactivated upon immunosuppressive treatment (Ramakrishnan, 2013). The model has provided several insights into the understanding of tuberculosis infections. For example, understanding the role of granuloma in pathogenesis (Swaim et al., 2006; Davis and Ramakrishnan, 2009; Ramakrishnan, 2013), role of bacterial eﬄux pumps in drug resistance (Adams et al., 2011), role of LTA4H in mediating inflammation against mycobacteria (Tobin et al., 2010), induction of necrotic death of infected cells mediated by TNFα (Roca and Ramakrishnan, 2013) and to study the manipulation of macrophage recruitment by mycobacteria (Cambier et al., 2014). Several therapeutic strategies against tuberculosis were employed or underway based on the insights from Zebrafish model (Torraca et al., 2014). In the current study, we used Zebrafish model to elucidate the role of rZmp1 in the dissemination of *M. marinum* and we have observed that upon injection of TdM. marinum mixed with rZmp1 resulted in dissemination of TdM. marinum to the tail region compared to the TdM. marinum injected alone or with heat-denatured rZmp1 (**Figures 6A–C**). Based on our quantitative analyses, we could infer that the presence of rZmp1 not only helped dissemination of TdM. marinum to the tail region per fish but also the number of fishes showing increased dissemination to the tail region were high (**Figure 6C**). Overall, the observations confirmed the possible role of rZmp1 in dissemination of *M. marinum* in Zebrafish.

We had earlier shown that Zmp1 is secreted by the virulent *M.tb* strain H37Rv in granuloma-like stress conditions (Vemula et al., 2016). With the present observations on rZmp1 inducing necrosis and the release of chemotactic chemokines from macrophages, we propose the following hypothesis that Zmp1 when released by mycobacteria in granuloma, may lead to both necrotic damage of the cells and release of chemotactic factors from surrounding infected macrophages. This would attract the uninfected immune cells like monocytes, dendritic cells and lymphocytes toward the site of necrosis. These cells may then get freshly infected by *M.tb* released from necrotic cells, thus assisting mycobacterial dissemination. The study has added new dimension to the understanding of molecular basis of mycobacterial pathogenesis. One may further explore how the concentration gradient of Zmp1 is decisive in necrosis on one hand and also lead a possible systemic dissemination on the other or does secretion of Zmp1 into acellular milieu of granuloma causes granuloma dissolution owing to proteolytic activity of Zmp1 and hence may have possible role in reactivation. In-depth studies in these lines would help in designing new anti-mycobacterial strategies to confront the emerging problem of drug resistance in *M.tb*.

## Author Contributions

Conceived and designed the experiments: MHV, RG, RM, SB, and KC. Performed the experiments: MHV, RG, RM, SS, and KJ. Analyzed the data: MHV, RG, RM, SB, and KC. Contributed reagents/materials/analysis tools: SB and KC. Contributed to the writing of the manuscript: MHV, RG, RM, SB, and KC. We declare that all the authors have approved the article for submission, its contents, order of authorship.

## Conflict of Interest Statement

The authors declare that the research was conducted in the absence of any commercial or financial relationships that could be construed as a potential conflict of interest.

## Supplementary Material

FIGURE S1 **Caseinase activity and cytotoxicity on CHO cells using purified, endotoxin-free rZmp1. (A)** Purified rZmp1 protein or heat-denatured rZmp1 (+ represents ∼ 4.8 μg and HD represents heat-denatured rZmp1) was incubated with casein (+ represents 10 μg) at 37°C in 100 mM Tris pH8.0. Small black arrowheads show the Casein (lane 2) and cleaved product of Casein (lanes 3 and 4). **(B)** Bar graph represents the cytotoxicity of CHO cells exogenously treated with endotoxin-free rZmp1 as determined using MTT assay.

FIGURE S2 **Exogenous treatment of THP-1 with rRv0047c does not cause cytotoxicity.** Bar graph represents the cytotoxicity of THP-1 cells exogenously treated with endotoxin-free rRv0047c [50, 100 nM or Heat-denatured (HD)] as determined using MTT assay. rRv0047c did not show any cytotoxicity on THP-1 cells at the above mentioned concentrations. These concentrations were used for Boyden chamber assays. All the experiments were performed more than three times.

FIGURE S3 **Dissemination of *M. marinum* mixed with non-specific protein, rRv0047c, or residual concentrations of LPS in Zebrafish.** The box plot represents the percentage of dissemination of TdM. marinum to the tail region. One day post fertilization Zebrafish were injected with TdM. marinum either with buffer control (Untreated) or non-specific rRv0047c (100 nM or 100 nM HD-rRv0047c) or LPS (1.25 pg). The dissemination of TdM. marinum to the tail region was then observed under fluorescence microscope on 5th day post infection and fluorescence intensities were calculated using ImageJ software (refer materials and methods). All the experiments were performed more than three times. ‘n’ represents the number of fish used per category.
