# Supplementary figures and images for: Mycobacterium tuberculosis Zinc Metalloprotease-1 Assists Mycobacterial Dissemination in Zebrafish
Source: Front Microbiol. 2016 Aug 29;7:1347. doi: 10.3389/fmicb.2016.01347 (PMC5002425; doi:10.3389/fmicb.2016.01347)

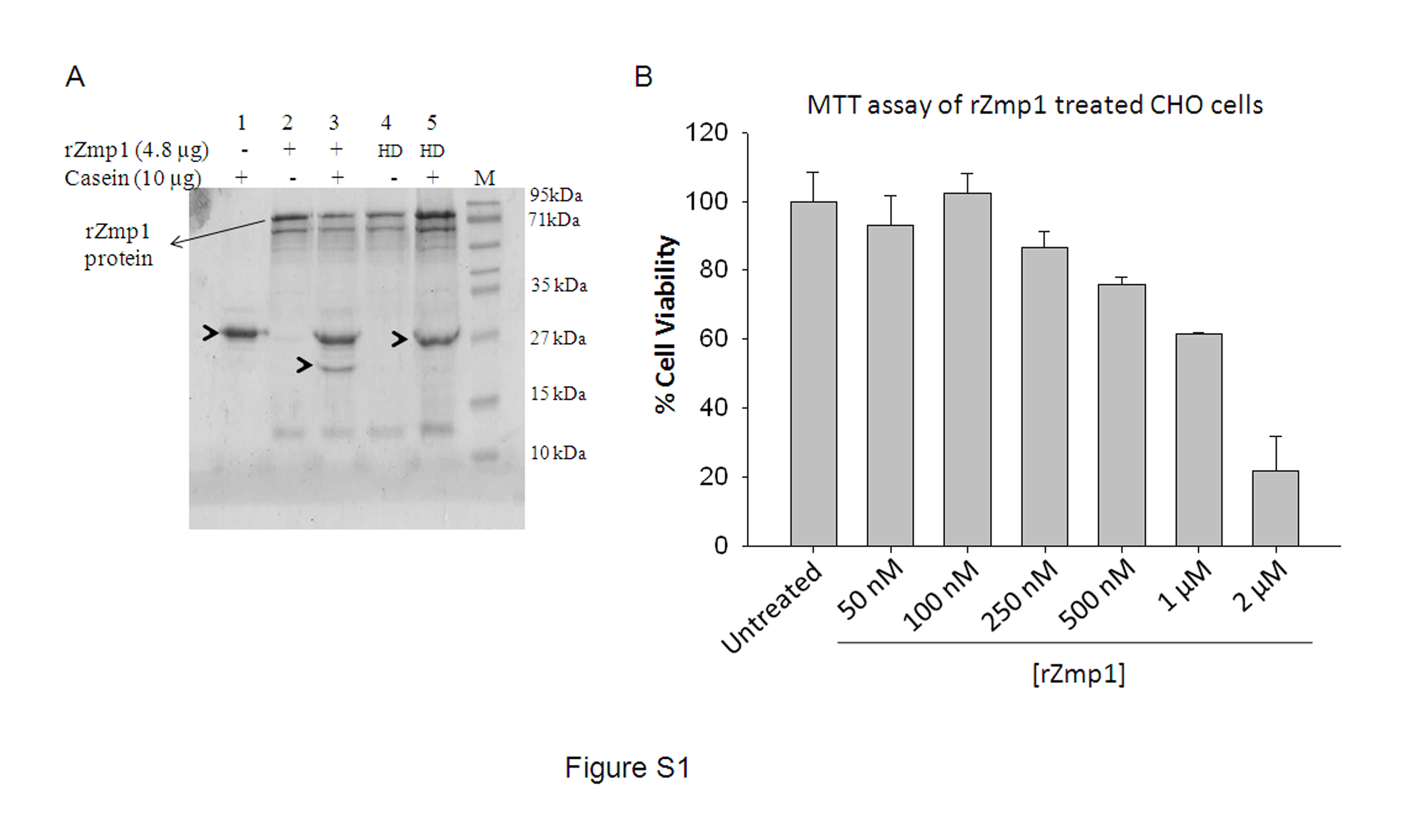

Supplement: FIGURE S1 — Caseinase activity and cytotoxicity on CHO cells using purified, endotoxin-free rZmp1. (A) Purified rZmp1 protein or heat-denatured rZmp1 (+ represents ∼ 4.8 μg and HD represents heat-denatured rZmp1) was incubated with casein (+ represents 10 μg) at 37°C in 100 mM Tris pH8.0. Small black arrowheads show the Casein (lane 2) and cleaved product of Casein (lanes 3 and 4). (B) Bar graph represents the cytotoxicity of CHO cells exogenously treated with endotoxin-free rZmp1 as determined using MTT assay. [file Image_1.TIF]

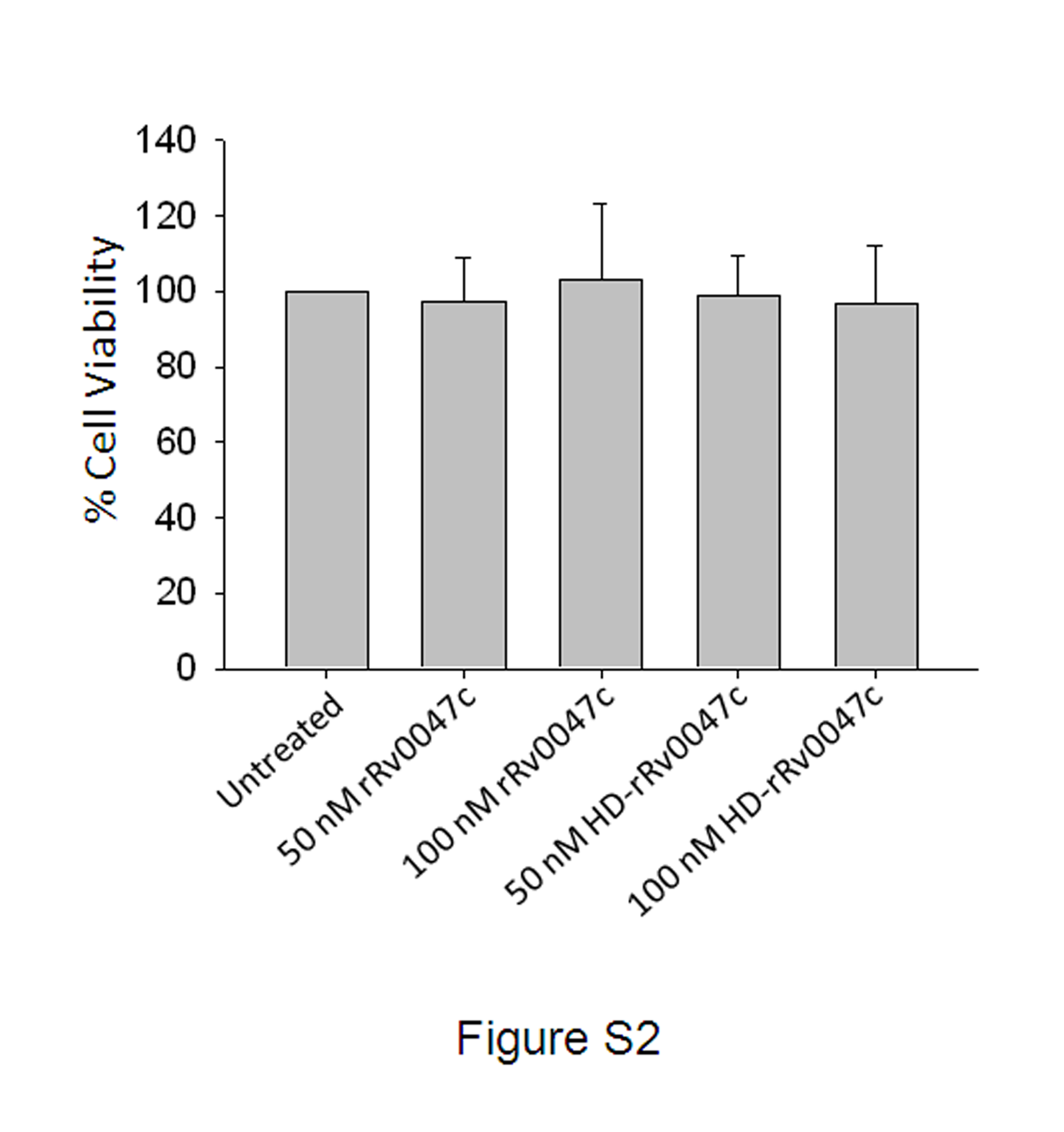

Supplement: FIGURE S2 — Exogenous treatment of THP-1 with rRv0047c does not cause cytotoxicity. Bar graph represents the cytotoxicity of THP-1 cells exogenously treated with endotoxin-free rRv0047c [50, 100 nM or Heat-denatured (HD)] as determined using MTT assay. rRv0047c did not show any cytotoxicity on THP-1 cells at the above mentioned concentrations. These concentrations were used for Boyden chamber assays. All the experiments were performed more than three times. [file Image_2.TIF]

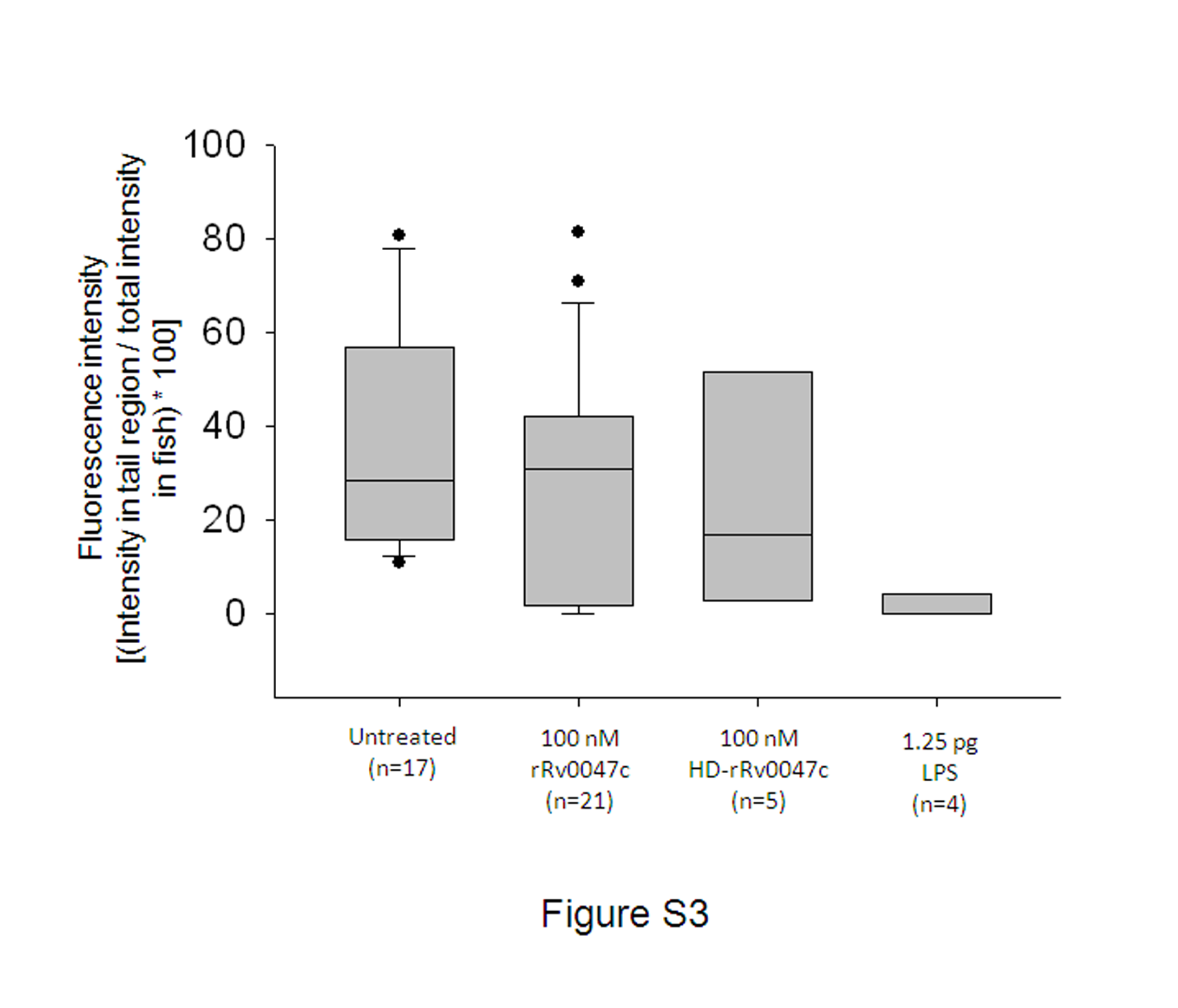

Supplement: FIGURE S3 — Dissemination of M. marinum mixed with non-specific protein, rRv0047c, or residual concentrations of LPS in Zebrafish. The box plot represents the percentage of dissemination of TdM. marinum to the tail region. One day post fertilization Zebrafish were injected with TdM. marinum either with buffer control (Untreated) or non-specific rRv0047c (100 nM or 100 nM HD-rRv0047c) or LPS (1.25 pg). The dissemination of TdM. marinum to the tail region was then observed under fluorescence microscope on 5th day post infection and fluorescence intensities were calculated using ImageJ software (refer materials and methods). All the experiments were performed more than three times. ‘n’ represents the number of fish used per category. [file Image_3.TIF]
